# A Giant Hepatic Hemangioma Presenting With Bilateral Pulmonary Embolism: A Case Report

**DOI:** 10.7759/cureus.24346

**Published:** 2022-04-21

**Authors:** Lama A Alharbi, Noora Abduljabbar, Mohammed Basheikh, Hind I Fallatah

**Affiliations:** 1 Medicine, King Abdulaziz University Hospital, Jeddah, SAU; 2 Medicine, Ibn Sina National College, Jeddah, SAU; 3 Medicine, King Abdulaziz University, Jeddah, SAU

**Keywords:** pulmonary embolus, inferior vena cava thrombus, hepatomegaly, hepatic hemangioma, case report

## Abstract

Hepatic hemangiomas are the most common benign tumors found in the liver. Herein, we present a case of a giant hepatic hemangioma (>4 cm) complicated by bilateral pulmonary embolism originating from the inferior vena cava thrombus with clinical and radiological features of portal hypertension. A 52-year-old woman presented to the emergency department of our institution with a history of blackouts. She underwent an extensive workup for potential causes of syncope, and a massive bilateral pulmonary embolism was detected. On examination, the patient was conscious, and her vital signs were within normal ranges. Abdominal examination revealed massive ascites and irregular nodular hepatomegaly without splenomegaly. Laboratory test results revealed normal liver function. The patient had elevated D-dimer levels. The serum-ascites albumin gradient was not elevated. Tumor marker levels were all within the normal range, and autoimmune profile results and test results for thrombophilia markers were negative. Abdominopelvic CT demonstrated hepatomegaly and a giant fungating chronic hepatic hemangioma occupying the right lobe along with an infrarenal inferior vena cava thrombus. The patient was discharged and prescribed a therapeutic dose of enoxaparin and diuretics. As the patient was not a candidate for resection due to the large hemangioma size and invasion of the liver tissue, she was referred to another center for a liver transplant. Hepatic hemangiomas are benign lesions and are usually managed conservatively since surgical intervention is controversial and is reserved for symptomatic or complicated cases. With an anatomically challenging lesion, enucleation/resection could not be achieved, and liver transplantation was the best achievable option.

## Introduction

A hepatic hemangioma is the most common benign lesion of the liver, with an estimated prevalence ranging between 0.4% and 20%. While the vast majority of hepatic hemangiomas are asymptomatic and discovered incidentally during radiological imaging, symptomatic lesions represent approximately 11% to 14% of cases [1,2]. Symptoms associated with giant hemangiomas (>4 cm) are usually attributed to pressure on adjacent structures [1]. Such pressure results in complications such as thromboembolic events secondary to inferior vena cava (IVC) compression [3]. Herein we report a case of a giant hepatic hemangioma, complicated by bilateral pulmonary embolism originating from an inferior vena cava (IVC) thrombus, with clinical and radiological features of portal hypertension.

## Case presentation

A 52-year-old woman presented to the emergency department at King Abdulaziz University Hospital (KAUH), Jeddah, on September 16, 2021, with a history of blackouts witnessed by her daughter, that lasted for a few minutes, and resolved spontaneously. The patient had no history of palpitations, shortness of breath, chest pain, abdominal pain, nausea, vomiting, or jaundice. We performed an extensive workup for potential causes of syncope, resulting in the diagnosis of a massive bilateral pulmonary embolism. The patient was diagnosed with a large hepatic hemangioma in 2011, after presenting with right upper quadrant abdominal discomfort. At that time, she was referred to a hepatic surgeon for hemangioma excision; however, the hemangioma was deemed unsuitable for removal due to the manner of hepatic parenchymal invasion. She underwent two sessions of radiofrequency ablation (RFA) as palliative treatment to reduce the symptoms of abdominal pain and discomfort. The patient was lost to follow-up until late 2016, when her disease state was found to have progressed to include ascites associated with the development of a large umbilical hernia. She presented to the emergency department at KAUH with leaking of ascitic fluid through the umbilicus. Surgical hernia repair was performed and the patient was subsequently discharged and prescribed furosemide 40 mg twice daily and spironolactone 25 mg twice daily. From then until early July 2021, her condition was stable with oral diuretics but without outpatient follow-ups. In the second half of July 2021, she stopped taking oral diuretics, resulting in recurrence of ascites that progressively worsened over time until her most recent admission in September 2021.

Physical examination

The patient was generally stable, not tachypneic, and was fully conscious with a Glasgow coma scale score of 15/15. Her mental status was normal, and she was very cooperative. She was afebrile with a body temperature of 36.5°C, pulse rate of 80/min, blood pressure of 122/77 mmHg, and oxygen saturation of 98% on room air, and she was not pale or jaundiced. We observed no stigmata of liver disease, such as flapping tremor, bruising, or spider nevi. Abdominal examination revealed severe distension, massive ascites, positive fluid thrill, irregular nodular hepatomegaly with a firm consistency, a liver span measuring approximately 22 cm, and no tenderness. No splenomegaly was observed.

Laboratory investigations

Laboratory test results showed that the patient’s liver function was normal. Complete blood count results showed normal white blood cell count, decreased hemoglobin concentration, and decreased platelet count. The international normalized ratio was elevated, and the prothrombin time was prolonged. The patient had elevated D-dimer levels, which suggested pulmonary embolism (Table 1). The serum-ascites albumin gradient was not elevated, measuring at 0.8 g/dL. Hepatitis C virus antibody and hepatitis B surface antigen test results were negative. Levels of tumor markers, including α-fetoprotein and carcinoembryonic antigen, were within normal ranges. The patient’s autoimmune profile and thrombophilia markers were tested; the results for anti-nuclear antibody, anticardiolipin antibody, B2-glycoprotein-IgG, and antimitochondrial antibodies were negative, and the serum ferritin level was within the reference range. The lupus anticoagulant test result was normal, and the patient’s smooth muscle antibody result was mildly positive. 

**Table 1:** Laboratory investigations WBCs: White blood cell count, Hb: Hemoglobin concentration, Plt: Platelet count, INR: International normalized ratio, PT: Prothrombin time

Laboratory Test	Result	Normal Range
Aspartate Amino Transferase	15 U/L	<34–118 U/L
Alanine Amino Transferase	<7 U/L	10–49 U/L
Albumin	42 g/L	40.2–47.6 g/L
Total Bilirubin	17 µmol/L	5–21 µmol/L
WBCs	7.8-× 10^3^/µL	4.5–11.5 × 10^3^/µL
Hb	10.8 g/dL	12–15 g/dL
Plt	134 × 10^3^/µL	150–450 × 10^3^/µL
INR	1.4	0.8–1.1
PT	15.6 s	11–13.5 s
D-dimer	>36.6 mg/L	0–0.5 mg/L

Radiological examinations

**Figure 1:**
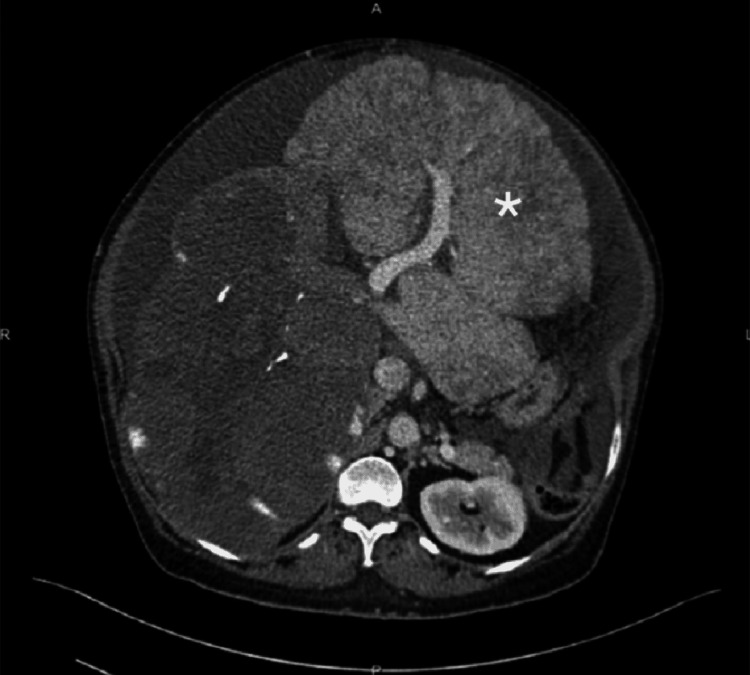
Axial non-contrasted CT image of the liver showing a giant fungating hemangioma (star).

**Figure 2:**
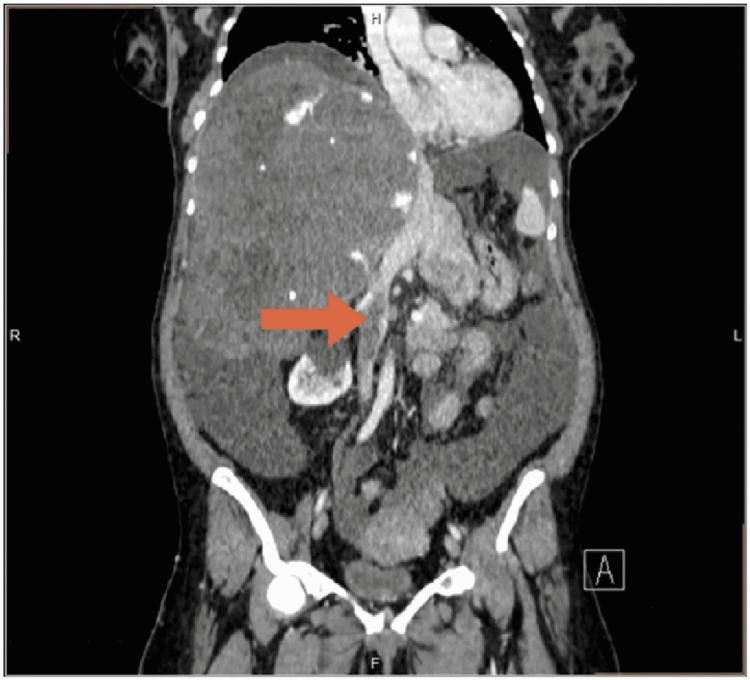
Coronal non-contrasted CT image showing the enlarged liver measuring 19 x 15 x 22 cm in cranial-caudal diameter and the IVC thrombus measuring 5.7 cm (arrow).

At her latest presentation on September 16, 2021, the patient underwent examination for the differential diagnosis of a pulmonary embolism and was found to have a typical electrocardiogram and CT findings. The CT pulmonary angiography showed acute main and lobar pulmonary arterial thrombi. Abdominopelvic CT demonstrated a giant fungating chronic hepatic hemangioma replacing the right lobe, with a central area of hypodensity and scattered macrocalcification along with a nodular enlarged liver measuring 19 × 15 × 22 cm in the cranial-caudal diameter (Figure 1). The main, left, and right portal veins were not clearly distinguished. The IVC and the left and right hepatic veins were compressed, and the middle hepatic vein could not be visualized. Images also indicated a new hemangioma developing on the left hepatic lobe, which was smaller in size and that was not noted on the previous CT reports. An infrarenal IVC thrombus measuring 5.7 cm in length was also visible (Figure 2). Doppler ultrasonography showed the main and left hepatic veins with no visualization of the right hepatic vein. Upper gastrointestinal endoscopy showed a normal esophagus without esophageal varices. Images from the post-RFA CT scan of the abdomen, which was performed one year after the palliative RFA, showed no size reduction in the hepatic hemangioma. A follow-up CT scan performed during the patient’s 2016 admission showed the development of moderate to marked ascites in the abdomen and pelvis with a lower abdominal wall hernia and no change in the size or appearance of the hepatic hemangioma. The patient was discharged and prescribed enoxaparin 60 mg twice daily and furosemide 40 mg daily. Her case was discussed by the tumor board committee, which included hepatologists, liver surgeons, and radiologists. Their consensus was that the patient was not a candidate for resection due to the large hemangioma size and invasion of the liver tissue, rendering it high-risk for resection. The patient was referred to another center for a liver transplant.

## Discussion

Hepatic hemangiomas are common benign liver lesions with a higher prevalence among females [4]. Most are discovered incidentally in asymptomatic cases, and in a minority of cases, the tumor grows, causing pressure on the adjacent structures, including the IVC, bile duct, hepatic vein, and portal vein. Moreover, vascular disturbances occur through several mechanisms, leading to complications such as pulmonary embolism [5,6]. Our report details the case of a patient with a giant, non-resectable hepatic hemangioma necessitating liver transplantation. However, until transplantation is achievable, the patient will be maintained on anticoagulants. A similar case with multi-segmental and perihilar liver involvement was reported, for which surgical intervention could not be accomplished, and the patient was administered anticoagulation for a long-term with warfarin [7]. Our patient had a pulmonary embolism with no evidence of lower limb deep venous thrombosis; however, hepatic hemangioma presenting with two episodes of deep venous thrombosis surgically treated by enucleation has previously been described [5]. A hepatic hemangioma >4 cm in size is termed giant and is presumably symptomatic. Pain in the right upper quadrant represents the most common symptom. However, other symptoms have been reported including nausea, vomiting, dyspepsia, and early satiety [2,8]. Our patient was evaluated for etiologies of thrombophilia such as autoimmune disease or hypercoagulability; she had radiological evidence of Budd-Chiari syndrome with supportive clinical symptoms, including abdominal pain and ascites. Hepatic hemangiomas are usually managed conservatively as surgical intervention is controversial and is reserved for symptomatic or complicated cases. Depending on the tumor size and location, evaluation by a multidisciplinary team is required to consider surgical intervention [6]. In our case, palliative RFA failed to reduce the tumor size, and enucleation was not possible. Therefore, liver transplantation, which has successfully been performed for non-resectable hemangiomas with an overall good outcome, represents the best available option [9].

## Conclusions

Hepatic hemangiomas are common and usually follow a benign course. Conservative management is usually appropriate for non-progressing and asymptomatic cases. However, in a small number of cases, hemangiomas grow to a large size (>4 cm), leading to complications such as symptomatic IVC compression and thrombosis. Surgical intervention is warranted in such cases to prevent severe complications.
